# Optimizing antimicrobial stewardship during operational upheaval: lessons in resiliency from the COVID-19 pandemic

**DOI:** 10.1017/ice.2026.10415

**Published:** 2026-05

**Authors:** Rebecca J. Schwei, Helena Ikenberry, Meggie Griffin, Nicole Werner, Lucas Schulz, Aurora Pop-Vicas, Ashleen Kaur, Sarah Scalzo, Michael S. Pulia

**Affiliations:** 1 BerbeeWalsh Department of Emergency Medicine, University of Wisconsin-Madison School of Medicine and Public Health, Madison, USA; 2 Department of Anesthesiology, Vanderbilt University Medical Center, USA; 3 Cepheid, USA; 4 Division of Infectious Diseases, Department of Medicine, University of Wisconsin-Madison School of Medicine and Public Health, Madison, USA; 5 University of Wisconsin-Madison, Madison, USA; 6 Department of Industrial and Systems Engineering, College of Engineering, University of Wisconsin-Madison, Madison, USA

## Abstract

**Introduction:**

The COVID-19 pandemic caused unprecedented operational stress on hospital-based antimicrobial stewardship programs (ASP). We utilized a systems engineering framework to characterize multi-level systems challenges to and strategies for resilient, hospital-based antimicrobial stewardship (AMS) during the COVID-19 pandemic.

**Methods:**

Using a national data set, we identified hospitals that had significant COVID-19 burden. We conducted semi-structured interviews with pharmacists, physicians and quality leaders involved in ASPs during the pandemic at those hospitals. Interview guides were developed using the Systems Engineering Initiative for Patient Safety (SEIPS) framework. Transcribed interviews were analyzed using deductive content analysis.

**Results:**

We interviewed 37 participants from 22 different healthcare systems across the country. Challenges to resilient ASP included physician employment model; limited AMS resources; staff shortages due to illness; shift in priorities; increased workload; remote work; and therapeutic momentum. Preexisting strategies to promote resilient AMS included system-wide AMS; decentralized AMS; excellent interprofessional relationships; strong culture of AMS and embracing incremental change. Real-time response strategies included ability to prioritize well; consistency with AMS work; being flexible and adopting change; intensifying infectious disease engagement; dedication to the profession; and reliance on automated tools and technology.

**Conclusion:**

Using a systems engineering informed qualitative approach, participants identified many modifiable challenges to AMS resiliency. Given the unfortunate reality that infectious disease pandemics and periods of operational stress are likely to occur in the future, we recommend that healthcare system leadership utilize the preexisting and real-time response strategies identified in this manuscript as a roadmap to ASP preparedness and a more proactive future response.

## Introduction

Suboptimal utilization of antibiotics in healthcare settings is a key, modifiable driver of the 2.8 million antimicrobial-resistant (AMR) infections and 35,000 associated deaths that occur in the United States annually.^1^ In 2016, the Joint Commission developed standards requiring hospitals to implement antimicrobial stewardship programs (ASPs).^2^ The COVID-19 pandemic was an unprecedented stress test for ASPs in all healthcare settings and significantly accelerated the overuse of antibiotics for viral acute respiratory infections.^3–8^ Unfortunately, ASP efforts were often deprioritized during the pandemic.^9,10^

With the re-emergence of Mpox, Marburg, and avian influenza^11–13^ local pandemics continue to present a significant future risk to routine ASP operations. By studying how ASPs responded during the COVID-19 pandemic, we can be better prepared to maintain essential components in times of operational stress. Therefore, guided by the Systems Engineering Initiative for Patient Safety (SEIPS), a systems engineering framework designed to evaluate healthcare work systems,^14^ we characterized multi-level systems challenges to and strategies for resilient, hospital-based antimicrobial stewardship (AMS) during the COVID-19 pandemic.

## Methods

### Sampling

We conducted semi-structured interviews with physicians, pharmacists and quality leaders involved with ASPs from hospital systems across the United States. We identified hospitals for recruitment using the Premier Healthcare Database, a deidentified database of approximately 25% of annual inpatient admissions in the United States.^15^ To be eligible, hospitals must have experienced a significant COVID-19 burden, and participants must have worked at the hospital during the COVID-19 pandemic (March 2020 to May 2023). To define COVID-19 burden, we determined the proportion of non-elective admissions with a COVID-19 diagnosis code (U07.1) and ranked hospitals. Eligible hospitals needed to be in the top 50% of COVID-19 burden.

Once eligible hospitals were identified, a Member Engagement and Recruitment Specialist at Premier connected us with ASP pharmacists and Infectious Diseases (ID) physicians and we confirmed eligibility via email. To ensure perspectives from sites outside of the Premier network, additional national leaders in ASP were recruited by the senior author (MSP) via direct email. We selected participants by purposeful criterion sampling to ensure we had representation based on roles (pharmacist vs physician); geographic region, and years of experience.^16^ Interviews and analysis occurred from January 2023-September 2024. The institutional review board approved all study activities. We followed the consolidated criteria for reporting qualitative studies (checklist in supplementary material).^17^ All participants received a financial incentive following the interview.

### Design and procedure

We conducted semi-structured interviews to explore broad themes around challenges to and strategies for resilient AMS. We defined resilient AMS as the ability of an ASP to anticipate, absorb, adapt to, and recover from disruptions while continuing to function effectively. Interview questions were primarily open ended so participants could respond freely. Follow up questions were based on elements of the SEIPS framework (interview guide in supplementary material). SEIPS is a systems engineering framework that conceptualizes healthcare delivery as a sociotechnical system, incorporating interactions among people, tasks, tools and technologies, organizational factors, and the physical environment to evaluate care processes and outcomes.^14^

A non-clinical, female study team member with 10 years of qualitative research experience (RJS) conducted the virtual video interviews in a private room. The principal investigator, a male emergency medicine (EM) physician with advanced training in systems engineering and qualitative methods (MSP), attended the first five interviews to observe, ask clarifying questions and refine the interview guide. Other female members of the research team with varying levels of experience (HI, MG, SS, AK) sat in on the interviews as notetakers and to ask clarifying questions. We refined the interview guide after three pilot interviews and continued to make minor adjustments as interviews progressed.

All interviews were audio recorded, professionally transcribed verbatim and reviewed for accuracy. We collected demographic (sex, role, board certifications, years of experience, tenure at current site) and geographic region (Northeast, South, Midwest, West) variables. Sampling, data collection, and data analysis occurred concurrently and data collection was stopped when responses became redundant and probes failed to uncover new themes (i.e. conceptual saturation).^18^

### Analysis

We used deductive directed content analysis guided by the SEIPS model.^19^ The study team developed a preliminary codebook based on post interview memos, interview question domains and elements in the SEIPS model.^20^ RJS coded all interviews and one additional study team member (HI, MG, AK, SS) independently coded each interview such that two study team members coded each interview. Coders met to review codes, add new codes and refine code definitions (codebook in supplementary material). As coding progressed, we grouped similar concepts into overarching themes. Discrepancies were resolved by discussion and consensus.^21^ We used Dedoose software to organize coding.^22^

## Results

**Figure 1.**
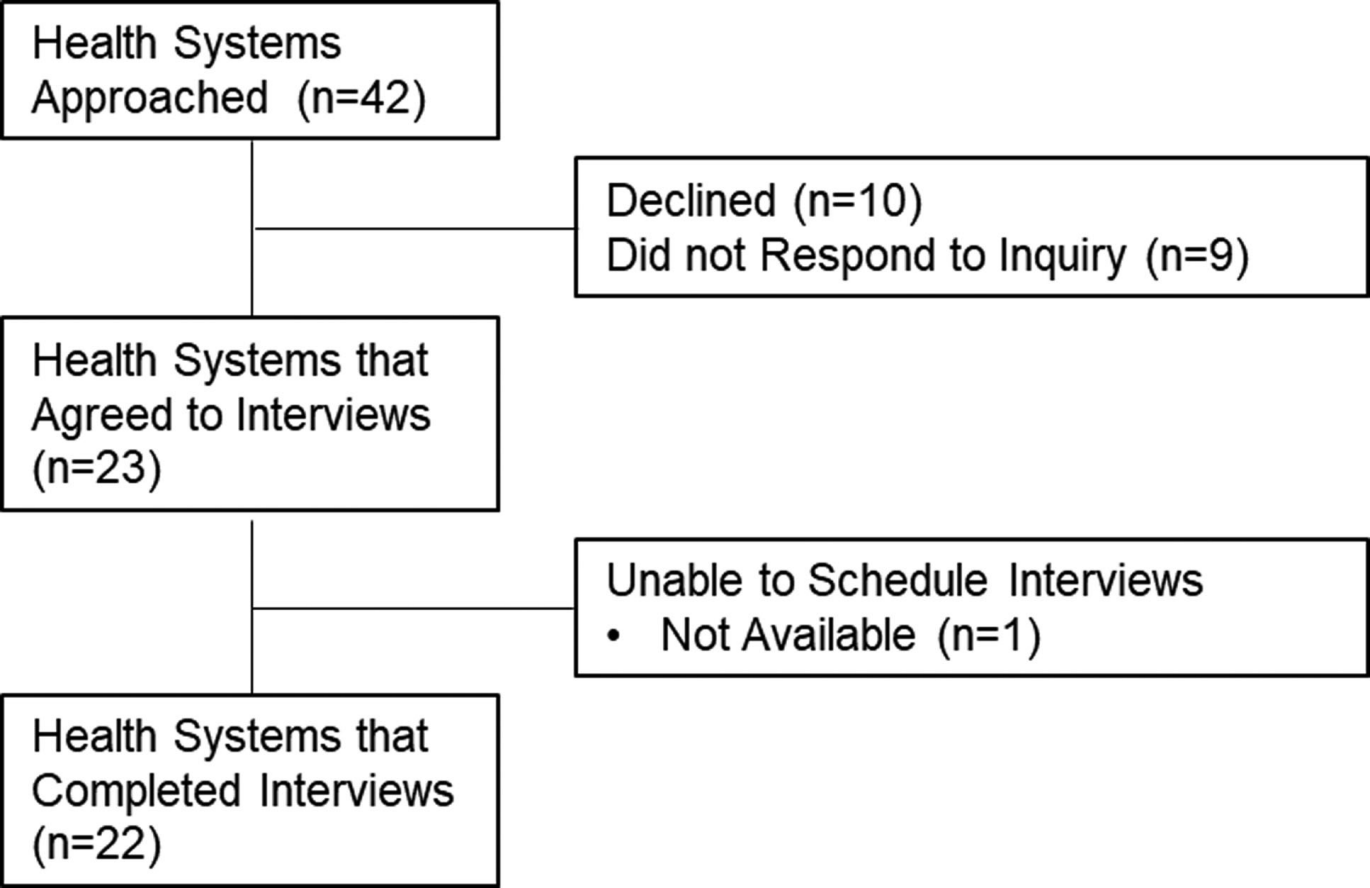
Flow diagram of recruitment.

**Figure 2.**
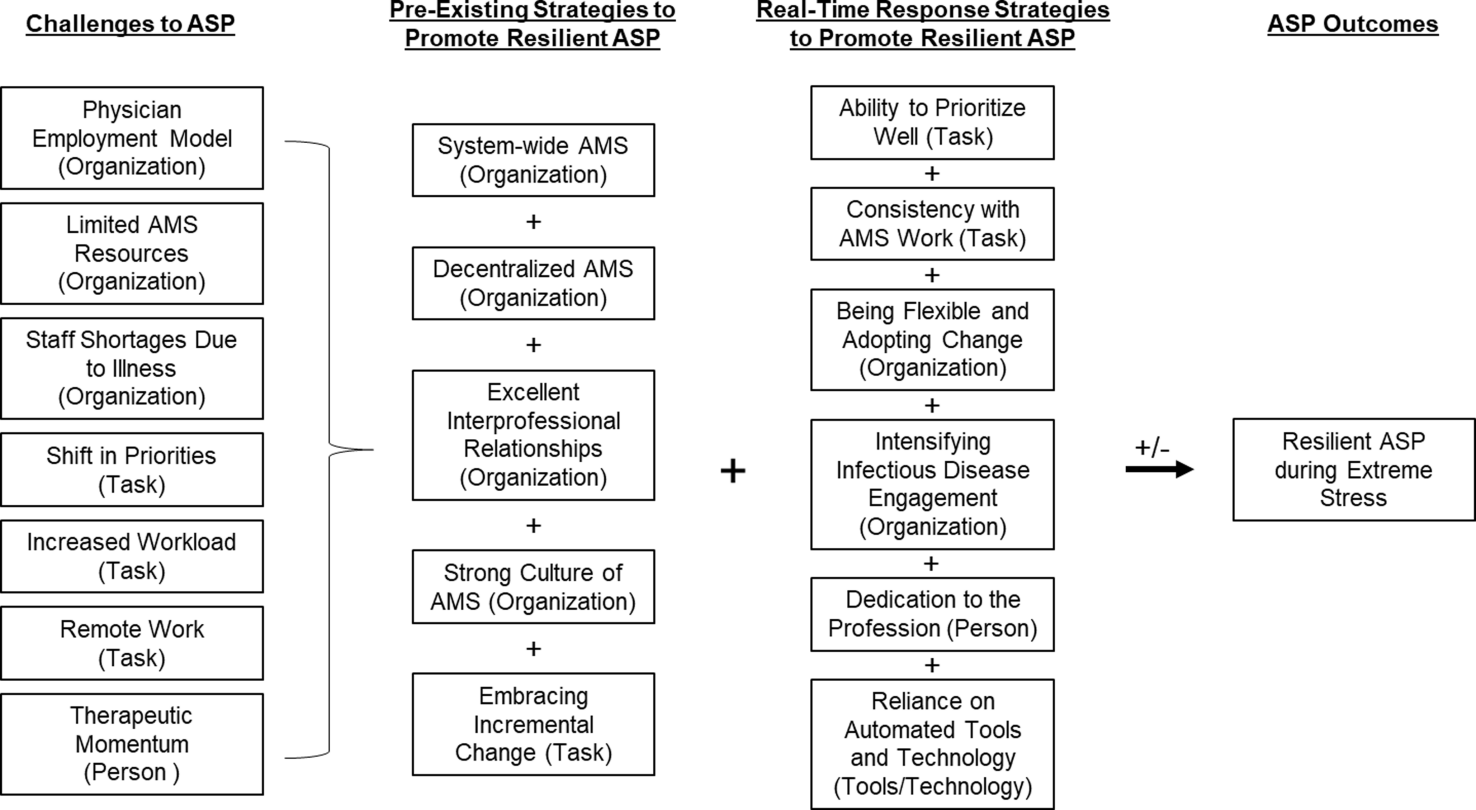
Description of challenges to antimicrobial stewardship programs that arose during COVID-19 pandemic and how pre-existing and real-time strategies were used to promote resiliency in ASP during the COVID-19 pandemic. SEIPS work system elements are in parenthesis behind each challenge or strategy. Abbreviations: AMS, antimicrobial stewardship; ASP, antimicrobial stewardship program.

We completed 36 interviews with 37 participants from 22 different healthcare systems (Figure 1). Table 1 describes participant sex, clinical role and the region of the country. Figure 2 is a diagram of the challenges and strategies to ASP resilience presented in the following results sections.

**Table 1.** Demographic and interview characteristics of interviews, *n*(%)

# Interviews	36
# People interviewed	37
# Systems	22
Clinical role	
Pharmacist	24/37 (64.9)
ID physician	8/37 (21.6)
EM physician	2/37 (5.4)
Hospitalist	1/37 (2.7)
Family medicine physician	1/37 (2.7)
IP nurse	1/37 (4.2)
Board certifications*	
Pharmacy - BCPS	12/37 (32.4)
Pharmacy- BCID	15/37 (40.5)
Pharmacy - none	1/37 (2.7)
Medicine- ID	9/37(24.3)
Medicine- internal medicine	7/37 (18.9)
Medicine- emergency medicine	2/37 (5.41)
Medicine- family medicine	1/37 (2.7)
Clinical informatics	2/37 (5.4)
Certified infection prevention	1/37 (2.7)
Mean years since training (range)	14.2 (1, 36)
Mean years at current site (range)	11.0 (1, 33)
Male	22/37 (59.5)
Region of the country by system	
Midwest	9/22 (40.9)
Northeast	5/22 (22.7)
South	6/22 (27.3)
West	2/22 (9.1)
Mean length of interview in minutes (range)	52.1 (42,69)

*Some participants are represented in more than one category. BCID, board certification in infectious diseases pharmacy; BCPS, board certified pharmacy specialist; ID, infectious diseases; EM, emergency medicine; IP, infection prevention.

### Challenges to ASP during the COVID-19 pandemic

Representative quotes for challenges to ASP during the pandemic are numbered C1 through C17 (Table 2).

**Table 2.** Challenges to resilient antimicrobial stewardship programs during the COVID-19 pandemic and representative quotes

Quote #	Category (work system element)	Participant role	Excerpt	Interview #
C1	Physician employment model (Organization)	ID Physician	This is a big issue because [physician’s not employed by the hospital] are going to do whatever. It’s not like they are employed by the hospital, and then we can do a protocol that’s applied, that is done and applied for everybody. We cannot do that because they do not work for the hospital here.	2022–2
C2	Physician employment model (Organization)	Pharmacist	We have some private practice docs, and we have some docs that are employed through the healthcare system. And sometimes you just have your more private practice docs that are a little bit less antimicrobial stewardship-focused because they’re not necessarily operating under the exact same umbrella as us, if you will. You know, they’re not going to like the stewardship meetings, or they’re, might not be as invested, I guess is the word I would say in some of those stewardship opportunities.	1016–1
C3	Limited AMS resources (Organization)	Pharmacist	There are certain aspects that we would love to do and we can’t fully accomplish, just based on our size and the number of FTEs or people or personnel that we have.	1002–1
C4	Limited AMS resources (Organization)	Pharmacist	We were really sort of patching things together with not a full lineup, our bullpen was empty, honestly. So, I think that made the management of these patients more difficult than maybe it could have been if the powers that be, again, this is administration, and certainly above my call, but if we had not cut back on some of our resources during that time.	1006–2
C5	Staff shortages due to illness (Organization)	Pharmacist	Probably the biggest impact was when our staff got COVID and had to be out for five to seven days, and we had to find coverage for them.	2022–1
C6	Staff shortages due to illness (Organization)	EM Physician	There was a time when even an exposure meant that you were quarantined for some period of days which led to tremendous issues in staffing	1001–1
C7	Sift in priorities (Task)	Pharmacist	But since it’s a period where we were so stressed out, we focused on the main things. Definitely, antimicrobial stewardship is very important, but there were other things that we needed to focus on in terms of operation, in terms of clinical work, and making sure that patients are getting the proper other therapies too.	2022–2
C8	Shift in priorities (Task)	Pharmacist	And our focus shifted from antimicrobial stewardship to now we’re in this COVID kind of stewardship, I guess, you would say.	2020–1
C9	Increased workload (Task)	Pharmacist	And it was more hours. It was more intense hours, because we were committed to keeping our core stewardship activities going. But then we had to superimpose on top of that the guidelines.	1011–2
C10	Increased workload (Task)	Pharmacist	The census was, oh, my God, I had days that my report was 100 pages. And I would think to myself, how can I get through 100 pages of reports, 100 plus? I mean, it was mind boggling. I had these moments of meltdowns. I remember being in my boss’s office like, I can’t do this anymore.	1008–1
C11	Increased workload (Task)	ID Physician	But obviously, everybody worked more clinically too. It was such a high volume of patients. And for our hospital at any given time, during the first surge, 80% of people had COVID. So we were all essentially rounding on probably 50 to 60 patients, each of us, as opposed to a normal volume of 15 to 20.	1015–1
C12	Increased workload (Task)	ID Physician	There was a huge burden on infectious disease. And I think it’s actually not just the question of being the frontline providers, because there were tons of frontline providers. It’s the constant, you know, here’s an article. What do you think about this, infectious disease provider? What about vaccination, infectious disease provider? What about this new medication? Should we include it in the treatment algorithm? So it’s the constant bombardment of responsibilities outside of being a frontline provider that, I think, was really challenging.	1007–1
C13	Remote work (Task)	Pharmacist	I think I could’ve been a better leader on what we should do, because everyone was in a panic on what to do, and everyone wanted to do something for our patients. And we didn’t know what to do. And I think if I could look back, I think our stewardship team, it would’ve been better for us to be more present to reassure providers that supportive care was the best we can do, and it would probably minimize harm to patients.	1005–1
C14	Remote work (Task)	Pharmacist	I wish we could’ve been out there on the floors. I mean, we were also sort of scared, like everybody was. So, I wish we could’ve also been out there and continued to show our faces and maintain those direct relationships and those face-to-face relationships with providers while this was all ongoing. Because I feel like that sort of thing would contribute to an increased intervention acceptance rate, and certainly, would potentially have curbed, that vancomycin and cefepime use in the setting of that chaos that was going on. If you had had that reassurance, that reassuring face, that personality to be there to kind of walk them back off that ledge.	1006–2
C15	Therapeutic momentum (Person & tools/technology	Pharmacist	A lot of the hospitalist services are just continuing what was started in the ED so, even though it’s tricky and patients move fast and there are throughput issues, whatever, we have found it productive to do as much as we possibly can in the ED because it often sort of sets the stage for what happens the next could of days of the hospital admission.	1012–2
C16	Therapeutic momentum (Person & tools/technology	ID Physician	One of the biggest challenges was related to the therapeutic momentum that came from the emergency room in that antibiotics were started and inpatient providers felt very nervous about stopping something that had been started in the emergency room.	1007–1
C17	Therapeutic momentum (Person & tools/technology	Pharmacist	Yeah, the ED will start them for like pneumonia mainly coming from the community. And then if the COVID test comes back positive, they initiate remdesivir. But they’re not so quick to DC the antibiotics once they’re initiating the remdesivir.	1016–1

#### Physician employment model (organization)

Physician employment model was defined as instances when physician employment (i.e. hospital employees vs private practice) impacted AMS work. C1 described how private practice physicians employed by their system made it challenging to implement an AMS protocol for all employees. In C2, a pharmacist described how private practice physicians are not as invested in AMS because they are not under the umbrella of the hospital.

#### Limited AMS resources (organization)

This included baseline limitations and limitations of resources during COVID-19. In C3, a pharmacist described how prior to the pandemic they could not do everything that they would like due to the size of their ASP. Furthermore, one pharmacist described how their ASP resources were cut during the pandemic (C4).

#### Staff shortages due to illness (organization)

Staff shortages due to illness or exposure were organizational challenges that arose during the COVID-19 pandemic. A reduced number of staff made it difficult to maintain AMS activities during the pandemic (C5, C6).

#### Shift in priorities (task)

Participants described shifting their priorities due to the COVID-19 pandemic. For example, in C7, one pharmacist acknowledged that AMS is important, but during a pandemic there were more important things to focus on such as ensuring patients received proper COVID-19 therapies. Likewise, in C8, one pharmacist described how her role changed from AMS to COVID-19 stewardship.

#### Increased workload (task)

This was a major challenge to maintaining AMS work. C9 described how in addition to their normal work, ID pharmacists were tasked with developing COVID-19 guidelines. Participants described how a high hospital census made it difficult to keep up with routine AMS work for pharmacists (C10) and physicians (C11). Finally, in C12, one ID physician described how in addition to being a frontline provider, ID physicians were expected to be available for frequent, informal consultations about patients, vaccines and treatments.

#### Remote work (task)

Pharmacists felt that antibiotics were overutilized during the pandemic was in part because AMS staff were required to work remotely. In C13, the pharmacist wished that he had been a stronger advocate which would have been easier had he been physically present. Likewise, in C14 another pharmacist described how being physically present may have led to increased intervention acceptance rates.

#### Therapeutic momentum (person)

Therapeutic momentum was defined as scenarios where it was hard to stop antibiotics once they were initiated. C15 and C16 described how it is hard for AMS pharmacists to stop antibiotics that are started in the emergency department (ED). The pharmacist in C17 said that even when a positive COVID-19 test resulted, providers would be quick to start COVID-19 therapy but would not stop antibiotics.

### Strategies for promoting resilient ASP during the COVID-19 pandemic

Participants described strategies for resilient ASP in two categories, preexisting and real-time response strategies.

#### Preexisting strategies

Preexisting strategies were in place prior to the pandemic. Representative quotes are numbered PES1 through PES15 (Table 3).

**Table 3.** Pre-existing strategies for resilient antimicrobial stewardship programs during the COVID-19 pandemic and representative quotes

Quote #	Category: sub-category (Work system element)	Participant role	Excerpt	Interview #
PES1	System-wide AMS (Organization)	Pharmacist	… sometimes when you can defer to something at, oh, this is what the system wants us to do, then it gets a little less personal when you’re having to enforce those guidance.	1012–1
PES2	System-wide AMS (Organization)	Pharmacist	We benefit greatly from the larger organization, yes…for us, it does, because, like I said, we don’t have the specialties. We don’t have ID. We don’t have anything like that, so it gives us access to a much wider knowledge base with some expertise.	1019–2
PES3	System-wide AMS (Organization)	ID Physician	Because if you’re just trying to operate based on the local observations, it’s not enough. And we didn’t have enough staffing for people to pull all the literature and look at all the data and review it on a committee level. So, the national committee was very valuable in terms of being able to generate that data.	1015–1
PES4	Decentralized AMS (Organization)	Pharmacist	…in the early days, when I first got my position, I was doing all these alerts. But then what we did is our decentralized pharmacists, they actually get a lot of these alerts in their daily queue, and then they respond to them. And then my real role shifted to educating them on how to respond to those alerts and then acting as troubleshooting in case they didn’t know what to do, which often happens.	1004–2
PES5	Decentralized AMS (Organization)	Pharmacist	We are a team. So we all [respond to alerts], all the decentralized pharmacists. And we make sure that we, so I’m the leader, if they have any questions, they ask me, but everybody is trained to do some antimicrobial stewardship activities, so that it’s not left to one person.	2022–2
PES6	Excellent interprofessional relationships (Organization)	ID Physician	I think overall, we’re fortunate that relationships between physicians and pharmacists, providers and pharmacists are pretty strong for the most part. There’s obviously some exceptions. I’d say in general, we have a very collaborative atmosphere	1011–1
PES7	Excellent interprofessional relationships (Organization)	Pharmacist	We typically do make sure that we introduce ourselves and kind of integrate ourselves in with the incoming year. So last year, we did do like dedicated rounds in the beginning of the year to meet all the new coming ID fellows and any new attendings that may have joined…I don’t think anyone would appreciate getting a call from someone telling them to do something different if they don’t have any kind of rapport.	2023–1
PES8	Strong culture of AMS (Organization)	ID Physician	I would say that we were pretty good because we were fortunate to have a pretty high-functioning stewardship program to begin with that had a lot of credibility. And so I think people were, yeah, I think people have always been very supportive in both to listen and they’re engaged. I’d say, you know, that’s why we’re a pretty high-functioning place.	1011–1
PES9	Strong culture of AMS: prior AMS success (Organization)	Pharmacist	Our stewardship team has a good track record of making things happen and had good data to support what we were doing. I think we made a good case that those activities were still valuable, and we needed to continue and actually double down on those and that we needed to pull in additional resources specifically dealing with COVID.	1005–1
PES10	Strong culture of AMS: prior AMS success (Organization)	Pharmacist	I think it was part of it was because we had emphasized viral, post-viral CAP and the need to not treat post-influenza, or to not treat influenza as a bacterial infection unless they were showing signs of post-influenza CAP, previously. But a lot of people were sort of willing to go along with this. This is a viral infection. We don’t need to give antibiotics to it.	1012–2
PES11	Strong culture of AMS: multidisciplinary buy-in (Organization)	Pharmacist	And then we do have, in our subcommittee, we do have like infection prevention, and we have quality. Our microlab person we’re in close contact with. Let’s see, microlab. We do have a nursing representative. We try to, we have our CMO, and we try to incorporate as many department heads as we can when we have those meetings, the provider and department heads. And any provider who is interested, we’ll invite them along, certainly. We love it when they want to use antibiotics appropriately.	1012–1
PES12	Strong culture of AMS: multidisciplinary buy-in (Organization)	Family Medicine Physician	We need to make a formal recommendation to limit antibiotics to the following cases. And we’d create a, it’s not really a protocol. We would create a guidance document and make sure everyone was on board with the guidance document. And then we would go ahead and try to educate and escalate the guidance document out to the system.	1004–4
PES13	Strong culture of AMS: ID engagement and leadership (Organization & person)	Pharmacist	I highly respect the physician who was here at the time … she was small but mighty. I mean, everybody really trusted her. She was like the go-to person for a lot of stuff in the hospital. Yeah. A really strong, she was really a strong leader. …She was, well, other than being just extremely intelligent, kind, yet just very to the point. Like, no, this is what you need to do, or not necessary.	1014–1
PES14	Strong culture of AMS: ID engagement and leadership (Organization & person)	ID Physician	I think we are fortunate to have a culture here where [ID] involvement is, for the most part, welcome. And where, actually, we often invite ourselves onto cases without even really being asked. Like specifically for things where, you know, we feel very strongly, but it’s, you know, for which there’s like evidence and literature to support superior outcomes for ID involvement.	1012–3
PES15	Embracing incremental change (Task)	ID Physician	It’s sometimes hard to say, especially if you’re not seeing a patient, and you’re just trying to intervene at a, stewardship level, it can be hard to say this patient does not need any antibacterial therapy. It’s easier to say this patient does not need a really broad spectrum antibiotic therapy that’s targeting multidrug resistant organisms.	2020–2

*System-wide AMS (organization).* We defined system-wide AMS as higher level coordination of AMS that promoted resiliency within hospital-level ASP. In PES1 one pharmacist described how having a system-wide stance on something helps prevent AMS conversations from feeling personal because you can defer to the system decision. Likewise, in PES2, system-wide AMS was helpful because he was from a critical access hospital and they did not have access to ID. Therefore, they relied on the system-wide AMS group to provide ID expertise. In PES3 an ID physician who was part of a national health system described how system wide AMS helped them generate meaningful data during a time of uncertainty.

*Decentralized AMS (organization).* Decentralized AMS included descriptions of AMS responsibilities being distributed across multiple pharmacists. In PES4, one pharmacist described how her role evolved from more of a centralized AMS model where the ID pharmacist responded to alerts to a decentralized model where the floor pharmacists were responsible for AMS alerts. In PES5 a pharmacist described how the decentralized AMS model works well in their hospital because it ensures AMS activities continue even if the ID pharmacist is out sick.

*Excellent interprofessional relationships (organization).* Excellent interprofessional relationships included descriptions of how positive working relationships with colleagues promotes AMS. In PES6 one ID physician described how they have a collaborative environment. Likewise, the pharmacist in PES7 described how they introduce themselves to new staff before they reach out about AMS recommendations.

*Strong culture of AMS (organization).* We defined a strong culture of AMS as descriptions of the hospital or system AMS mindset facilitating AMS work. For example, PES8 described their AMS group as highly functioning with a lot of credibility. Within this category, participants described three subcategories including prior AMS successes, multidisciplinary buy-in and ID engagement and leadership.

*Subcategory: Prior AMS success (organization):* Participants described how prior AMS success contributed to additional AMS resources and acceptance of COVID-19 treatment recommendations. In PES9, one pharmacist said that because of historically good AMS outcomes, they successfully advocated for additional resources during the pandemic to maintain AMS activities. Likewise, in PES10, the pharmacist described how the AMS program had previously been working on not treating viral infections with antibiotics, and therefore, during the pandemic providers were receptive to not treating COVID-19 infections with antibiotics.

*Subcategory: Building multidisciplinary buy-in (organization)*: Participants talked about the importance of involving a wide variety of disciplines in AMS processes and recommendations. In PES11, one pharmacist described how they invite anyone who is interested to their AMS sub-committee meeting. In PES12, a family medicine physician described how they incorporated multidisciplinary input to their COVID-19 treatment guidance.

*Subcategory: ID engagement & leadership (organization and person):* ID engagement and leadership contributed to strong AMS culture. In PES13 one pharmacist described how the ID physician at her hospital was a strong leader, and how this set the tone for AMS. PES14 described how the ID group was empowered to consult on cases involving antibiotic stewardship concerns which helps support the pharmacist’s AMS recommendations.

*Embracing incremental change (task).* We defined embracing incremental change as descriptions of how small gradual changes can make a difference. For example, in PES15, one ID physician described how it can be easier to convince physicians to narrow the spectrum of antibiotics versus discontinue antibiotics entirely.

*Real-time response strategies.* Real-time response strategies (RTRS) were implemented during the COVID-19 pandemic and promoted ASP resiliency. Representative quotes are numbered RTRS1 through RTRS14 (Table 4).

**Table 4.** Real-time response strategies for resilient antimicrobial stewardship programs during the COVID-19 pandemic and representative quotes

Quote #	Category: sub-category (Work system element)	Participant role	Excerpt	Interview #
RTRS1	Ability to prioritize well (Task)	Pharmacist	A lot of it too is prioritization. Even though we try to start with the ICUs first because they are more highly acute. But if the patient, we’ve already seen them on a daily basis, and they’re kind of like stable, even though you’re not really stable in the ICU, but you know what I mean? If what we’re treating is the same day in and day out, we move on faster, you know and then just give priority to patients that from just the looks on paper and the chart that they might not be on appropriate therapy.	1008–1
RTRS2	Consistency with AMS work (Task)	Pharmacist	I think that’s my most significant thing I learned, is just accept the fact that people will do a bunch of crazy stuff…just continue to try to do the small things that you all already do before the pandemic. And don’t let the pandemic stop you from what you’re doing, as far as the daily antimicrobial stewardship responsibilities because I think that ultimately, that is where you get most of your money from, doing the small thing over and over and over again.	2022–1
RTRS3	Consistency with AMS work (Task)	Pharmacist	What I think is most important for resiliency, not just in stewardship, but for everything, is that people know what they’re doing is making a difference… And we’re going to be faithful in what we’re doing every day. And if we’re faithful in what we’re doing, we think it’s going to show up in other ways.	1005–1
RTRS4	Being flexible and adopting change (Organization)	Pharmacist	[During the pandemic] we might implement things before it went to P&T…there was just so many changes happening so quickly, that it happened faster than what our P&T meetings would permit.	2024–1
RTRS5	Being flexible and adopting change (Organization)	Family Medicine Physician	And as I mentioned, we were able to meet fairly quickly. And we could make changes quickly. I think the infrastructure was very agile. We were able to move a lot quicker than, and make decisions, and get things produced a lot quicker than we normally would have.	1004–4
RTRS6	Being flexible and adopting change: increasing AMS staffing (Organization)	ID Physician	The health system was generous during that time in terms of, we were not able to hire anybody because it was just too chaotic to try and post a position and try and interview people. But, during like the second year of COVID, we were able to have another person to join our group to get us back up to appropriate staffing. And even during the third year too, we were able to bring, you know, another person on too and get us up to higher staffing.	1015–1
RTRS7	Being flexible and adopting change: increasing AMS staffing (Organization)	ID Physician	I think the things that maybe were helpful was after there was a cut, within about, let’s see, within about six to nine months after that cut, they actually reinstituted the funding. So we hired a new ID pharmacist.	1013–1
RTRS8	Intensifying ID engagement (Organization)	Pharmacist	But I think that ultimately, because there was such, so much unknowns, well, pretty much all patients got consulted [by ID].	2023–1
RTRS9	Intensifying ID engagement (Organization)	ID Physician	You know, you have a deadly disease, for which you don’t have a treatment. I mean it’s a viral disease, so throwing antibiotics straight up is not going to help. And it sort of, unless you model that, unless you show that, no one’s going to catch on, and no one’s going to see that. So you have to be the voice of reason. You have to be the voice of calm.	1007–1
RTRS10	Intensifying ID engagement (Organization)	ID Physician	It was our time, it was our time. It was like the 1st in a 100-year epidemic, pandemic. And ID was needed…It was our time to serve, it was our time to serve. It was what we had to do. And that’s what we did as a group, every one of us, including the faculty. People knew it was our time to step up because they could see a horrible tragedy that was occurring. And they just knew we just had to do it because there was no plan B… If we didn’t do it, nobody was going to do it.	1015–1
RTRS11	Dedication to the profession (Person)	Pharmacist	There were a lot of people who sort of made extraordinary efforts to have prescribing be the right way and therapeutics be the right way for us. But it was using the systems that were in place already. So I would say both of the like personal excellence and the prior systems and information sharing.	1012–2
RTRS12	Dedication to the profession (Person)	ID Physician	Now I was for stewardship from day one, and I decided to lean very heavily into that. To my detriment, perhaps, yes. But I quickly realized by March, April of 2020, that was going to be my fight. Was that every day was going to be a constant reminder of antibiotics don’t fix a virus.	1017–2
RTRS13	Reliance on automated tools and technology (Tools and technology)	Pharmacist	The module would be like anybody on antibiotics, and you could set up different columns. So there was like IV to PO. Like it would flag if there was a potential for that. It would flag if, you know, it saw potential for de-escalation, insufficient coverage, or bug-drug mismatch. So it would basically kind of find my interventions for me, and I picked which ones we thought were the highest impact.	1006–1
RTRS14	Reliance on automated tools and technology (Tools and technology)	Pharmacist	And we, what we did is we used our decision support software to let us know if a patient had a COVID test and was on antibiotics. And so then our team would evaluate the patient, and, you know, they could have another source of infection, maybe a urinary tract infection, and so antibiotics could be indicated. But if it was all respiratory and COVID, then we would try and focus on that.	1005–1

*Ability to prioritize well (task).* Ability to prioritize well captured how participants changed day-to-day work due to staff shortages and increased workload during the COVID-19 pandemic. For example, in RTRS1 a pharmacist described prioritizing higher impact aspects of antibiotic appropriateness review during the pandemic.

*Consistency with AMS work (task).* We defined consistency with AMS work as descriptions of how doing daily AMS tasks leads to improvements and resiliency. In RTRS2 and RTRS3 pharmacists described how even during a pandemic when things were chaotic, it was still important to maintain daily AMS activities.

*Being flexible and adopting change (organization).* Being flexible and adopting change included when institutions implemented new workflows or described the value of adopting organizational changes during the pandemic. In RTRS4, one pharmacist described how during the COVID-19 pandemic they were allowed to bypass the pharmacy and therapeutics committee approval to rapidly respond. Likewise, in RTRS5 a family medicine physician described how they made decisions and implemented workflows more quickly during the pandemic. Within this category, participants also described a subcategory: increasing AMS staffing to respond to the COVID-19 pandemic

*Subcategory: Increasing AMS Staffing (Organization)*: This strategy allowed ASP to absorb additional responsibilities. In RTRS6 one ID physician described how their health system allocated more people to the ID team because they were the group with the increased workload. Likewise, in RTRS7, an ID physician described how although at the beginning of the pandemic an ID pharmacist position was cut, after about 6 months they were given more staffing resources.

*Intensifying ID engagement (organization).* Another strategy participants described that promoted ASP resiliency was intensifying ID engagement. In RTRS8 one pharmacist described how due to the uncertainty around COVID-19, most patients received an ID consultation. Likewise, in RTRS9, one ID physician described how they viewed it as their role to be the ‘voice of reason’ and to help other providers understand that it was not appropriate to use antibiotics in a viral infection. Finally, in RTRS10 another ID physician described how because this was a pandemic, ID needed to step up and use their expertise for the greater good.

*Dedication to the profession (person).*We labeled something as dedication to the profession when participants described going above and beyond their normal duties because of an internal dedication to ID work. In RTRS11, one pharmacist described how throughout the hospital many ID professionals made extraordinary efforts in response to the COVID-19 pandemic. Likewise, in RTRS12, one ID physician described how he made a conscious decision to focus on AMS during the COVID-19 pandemic.

*Reliance on automated tools and technology (tools and technology).* Throughout the interviews, participants described relying heavily on automated tools during the COVID-19 pandemic. In RTRS13, one pharmacist described how they used an automated program during the pandemic to identify high priority AMS interventions. Likewise, in RTRS14, another pharmacist described how they had a decision support tool built for COVID-19 specifically to help their ASP.

### Discussion

Using a systems engineering framework informed qualitative approach, we characterized work system challenges to and strategies for resiliency among ASPs during the COVID-19 pandemic. The SEIPS model allowed us to consider both the hospital and ASP work systems so that we could identify challenges and strategies that extend beyond the patient and the provider.

Participants identified a variety of challenges to AMS resiliency during the COVID-19 pandemic. Three of the challenges, physician employment model, limited resources and therapeutic momentum, were challenges to ASP prior to the pandemic and continued to negatively impact ASP work during the pandemic. The other challenges, staff shortages due to illness, shift in priorities, increased workload and remote work, were new challenges that arose during the pandemic. Interestingly, the challenges to ASPs identified in this study were modifiable, suggesting they could be overcome by implementing the strategies described in this and other studies.^23–29^

In future pandemics, it is critically important to maintain AMS activities. We hypothesize that the deprioritization of AMS activities, which was reported in this study as well as in prior work,^9,10,30,31^ contributed to the overuse of antibiotics for viral acute respiratory infections^3–8^ and the subsequent increase in bacterial antimicrobial resistant hospital-onset infections.^32,33^ Prior work has highlighted how routine AMS activities such as prospective audit and feedback contributed to lower antibiotic use compared to usual care during the COVID-19 pandemic.^23,34^ ASP and ID professionals are needed to support appropriate use of antibiotics, and their role may be even more essential during a pandemic where high levels of stress may contribute to increases in inappropriate use of antibiotics.

It is in the best interest of health systems to incorporate lessons learned from COVID-19 to enhance preparedness and enable a proactive response.^25,35^ Re-emerging infectious diseases such as Mpox, Marburg, and avian influenza highlight the threat of a future pandemic.^11–13^ For ASPs, the preexisting strategies identified in this study included system-wide AMS, decentralized AMS, excellent interprofessional relationships, strong AMS culture and embracing incremental change. Four of these strategies are within the organization domain of the SEIPS model suggesting that the behavior of antibiotic prescribing is impacted by more than the patient and provider beliefs and attitudes.^26,36–39^

Participants identified real-time response strategies that allowed their ASP to function effectively during the pandemic. Intensifying ID engagement, prioritization and consistency of AMS work, and reliance on automated tools and technology were strategies participants utilized in response to limitations on resources such as personnel and time. These findings are supported by prior work which has suggested that additional AMS resources and enhanced utilization of tools and technology are helpful during pandemics to mitigate increased workload.^24,30,40–42^

Another important finding from this study was how personal dedication of the ID professionals throughout the pandemic contributed to ASP resiliency. This is an important finding for future periods of stress because healthcare leaders need to do a better job of recognizing the extraordinary efforts of individuals in real-time, providing resources and compensation to ensure this essential work is completed.^43^

The study results should be considered in the context of the study’s limitations. The first limitation to consider is selection bias both because the recruitment strategy was opt-in, and because our sampling frame only recruited staff from hospitals with significant COVID-19 burden. Second, due to elapsed time since the start of the pandemic, the fluidity of recommendations during the pandemic and the traumatic nature of the pandemic, participants’ memories were not always clear on how certain events transpired.

Using a systems engineering informed qualitative approach, we characterized work system challenges to and strategies for resiliency during the COVID-19 pandemic specific to the ASP work system. Participants identified challenges to AMS resiliency, many of which were modifiable and could be addressed in future pandemics. Healthcare system leadership should utilize the identified preexisting and real-time response strategies as a roadmap to ASP preparedness and an effective, proactive response to future instances of operational stress.

## Supporting information

Schwei et al. supplementary material 1 Schwei et al. supplementary material

Schwei et al. supplementary material 2 Schwei et al. supplementary material

## Supplementary material

The supplementary material for this article can be found at https://doi.org/10.1017/ice.2026.10415.
