# Supplementary material for: Optimizing antimicrobial stewardship during operational upheaval: lessons in resiliency from the COVID-19 pandemic
Source: Infect Control Hosp Epidemiol. 2026 Mar 26;47(5):499–508. doi: 10.1017/ice.2026.10415 (PMC13040247; doi:10.1017/ice.2026.10415)
Supplement: Schwei et al. supplementary material 1 — Schwei et al. supplementary material [file S0899823X26104152sup001.docx]

| **Work System Components** | | |
| --- | --- | --- |
| **External Environment:** Regulatory, legislation, medical/legal environment and other high level or societal factors that are external to healthcare system. Includes medical literature and national guidelines. Includes any reference to local bacterial resistance patterns including epidemiology of organisms and references to local geography culture or population density. Includes populations of people | | |
| **Internal Environment:** The physical environment in which the diagnosis and treatment decisions are made. This includes the emergency room or hospital layout, temperature, noise levels, crowding, observation unit, boarding patients | | |
| **Organization:** The larger context in which decisions surrounding infection management and antibiotic use are made. Including, the hospital culture, policies and procedures, work schedules, and relevant training provided. Local healthcare environment, treatment algorithms. Organization should be used for facility level policies or other higher level factors that influence decision making; how decisions are made among people; communication, collaboration and teamwork; how work is organized. | | |
| **Person- Healthcare Staff:** Characteristics including the education, skills, motivation and knowledge of the healthcare team, individual healthcare professional, that affects their performance. Can include descriptions/characteristics of a specific group of people more broadly, ED staff or nurses, etc. Person should be used when talking about individual people, groups of people. Includes relationships between providers | | |
| **Person- Patient:** Patient or family characteristics including education, skills, motivation and knowledge. Can include descriptions/characteristics of a specific group of people more broadly, patients. Person should be used when talking about individual people, groups of people. Includes relationships between patients | | |
| **Tasks:** The individual activities performed during or are associated with the diagnosis of infection, treatment of infection, utilization of antibiotic (e.g. evaluating the patient, deciding on a treatment, deciding on a disposition, administering the treatment, documenting the process in the EHR) | | |
| **Tools and Technology:** Anything the healthcare worker uses for the diagnosis and treatment such as diagnostic tests, supplies, imaging, external references. Antibiotics are not considered tools and technology; can include phones, computers, text-chat; spirit of tasks is coding the characteristics of tasks i.e. ask complexity, route task, fast task, cumbersome task. Includes when participants talk about day-to-day tasks shifting or not shifting | | |
| **Process** | | |
| **Conducting Antimicrobial Stewardship**: Describes process of doing antimicrobial stewardship tasks at their hospital or at their system. Can be pre-pandemic or during any phase of the pandemic | | |
| **Conducting Antimicrobial Stewardship for COVID-19 patients:** Describes the process of doing antimicrobial stewardship for COVID-19 patients or patients with known viral infections. This includes discussions surrounding antibiotic use for COVID-19, influenza, RSV patients | | |
| **Time Period** | | |
| **Pre COVID-19:** Participant is talking about the time before COVID-19. Includes when they are not specific about when things were happening | | |
| **During COVID-19:** Participant is talking about the time during COVID-19 or immediately after | | |
| **Challenges that Get in the Way of AMS** | | |
| **External Environment** | | |
|  | **Cost of Drugs or Tests:** Participants describe how the cost of drugs, or therapeutics or lab tests impacted what they did for AMS | |
|  | **Evolving Landscape:** Participants describe how an evolving landscape made it hard to do AMS. Could be supply shortages or other constant changes made it really difficult to work. Would also include how it was difficult to keep up with high volumes of literature or that as an institution we were very reactive | |
|  | **Knowledge Gaps:** Participants describe how limited knowledge or gaps in knowledges negatively impacts AMS; would also include conflicting statements or evidence. Would include comments about this being a new disease state | |
|  | **Lack of Therapeutics:** Participants describe not having treatments for COVID-19 or other diseases; would also be difficulty or challenges accepting that all we have to offer is supportive care. Would also include the need to do something | |
|  | **Sepsis Core Measures:** Participants were describing how the SEPSIS Core measure made it hard to use antibiotics appropriately in patients with COVID-19 | |
| **Organization- AMS Program Challenges** | | |
|  | **Centralized AMS:** A small group of people are responsible for all AMS activities, and they can't keep up. Includes the idea that one size doesn't fit all with AMS system activities | |
|  | **Limited Resources:** Participants describe how it can be hard to do AMS due to limited resources or not enough money or not enough time/bodies. Would also include pay cuts. Includes small sites not having ID expertise | |
|  | **Staff Shortages:** Participants describe staff shortages or the absence of certain types of staff during parts of the day (we only have a pharmacist during business hours etc.) | |
|  | **New/Shift in Responsibilities:** Participants describe how they had to shift their responsibilities and how that got in the way of AMS | |
|  | **Non-Coordinated Response**: Participants talk about how people were putting stuff out without stakeholder review (i.e. antibiotics on order set w/out ID review). Would also include lack of a system wide response or guideline | |
| **Organization – Hospital Challenges** | | |
|  | **Changes in Patient Population:** Participants describe how changes in the hospital patient population and how that impacts AMS activities | |
|  | **Effective Dissemination:** Participants describe challenges with effective dissemination of guidelines etc. | |
|  | **Hindering Provider Autonomy:** Participants talk about physicians or other providers being frustrated that their autonomy was being taken away through for example more of the structured AMS activities. Only being allowed to order Remdesivir through an order set, etc. | |
|  | **Hospital Employment Model:** Participants talk about the employment model of the hospital or having private practice physician groups gets in the way of AMS | |
|  | **ID Was Not Managing COVID-19 Patients:** Participants describe how ID wasn't involved in COVID-19 or how other provider groups took over management | |
|  | **Leadership Gaps:** Participants talk about how leadership vacuums contribute to issues with AMS. This could be specific to gaps in ID leadership or more broad to hospital leadership gaps; would include comments like, everyone was reacting | |
|  | **Low Morale:** Participants describe how there was low morale in the hospital. Doesn't matter the reason, and type of burnout, discouragement would count | |
|  | **Patient Volumes:** Participants describe how high or low volumes influence antibiotic prescribing patterns | |
|  | **Practice Variability:** Participants describe how variation in practice patterns makes it harder to do AMS | |
|  | **Remote Work/Social Distancing Requirements:** Participants describe how working remotely or not at the hospital where the patients are impacts AMS. Would include comments where the participants describe not being able to go out on the floors as much or not being able to meet in person to make decisions | |
|  | **Trouble Adapting to the Quickly Evolving Pandemic:** Participants talk about ways they struggled to adapt to an evolving pandemic. Successful systems were more flexible, these systems struggled to adapt | |
|  | **Turnover:** Participants talk about turnover that impacts AMS, could even be that we are a teaching facility so we have a lot of resident turnover etc | |
|  | **Waiting Times:** Participants describe how long wait times make them more likely to want to prescribe antibiotics | |
| **Person (patient) Challenges** | | |
|  | **Complexity of Patients**: Participants describe how the complexity of patients impacts AMS | |
|  | **Patient Request or Noncompliance:** Participants talk about the challenge of patient request for certain treatments or patients not being compliant about IP protocols. Would also include stated perception that patients needed the antibiotic to feel like they got some treatment | |
|  | **Patient Severity:** Participants describe how really sick patients impact AMS. Would also include comments of long length of stays or nosocomial infections impacts on AMS | |
| **Person (provider) Challenges** | | |
|  | **Diagnostic Uncertainty:** Participants describe how not knowing what the illness is or how not being sure if there is a bacterial coinfection impacts AMS | |
|  | **Differences of Opinion:** Participants describe how differing opinions impact AMS. This includes when some opinions are not evidence based | |
|  | **Fear:** Participants talk about healthcare staff being afraid of COVID-19 and having to overcome that | |
|  | **High Antibiotic Utilizers/Utilization:** Participants talk about certain providers or provider groups being high utilizers. Could also be about general high antibiotic utilization during COVID-19 or when antibiotics were included on an order set. This code should be used when there isn't a clear reason as to why people are using a lot of antibiotics. If they indicate the reason why then code it as that. | |
|  | **High Stress Levels and Overwhelmed Providers:** Participants mentioned providers being overwhelmed, burned out etc. and how this impacted AMS | |
|  | **Non-Evidence Based Recommendations:** Participants talk about how non-evidence based recommendations from other providers or the government was challenging | |
|  | **Not Familiar with AMS:** Participants describe certain providers or provider groups not understanding AMS or learning about it during training | |
|  | **Personal Pressure to Respond:** Participants describe personal pressure they were under during COVID-19 to provide leadership, determine who was getting treatments, etc. | |
|  | **Providers Did Not Want to Learn How to Manage COVID-19 Patients:** Participants talk about certain providers or provider groups not wanting to learn how to manage COVID-19 patients | |
|  | **Unwillingness to Accept AMS Recommendations:** Participants describe how some providers are not willing to accept AMS recommendations; would include unwillingness to follow findings of tests, viral panel results but still treating bacterial infection | |
|  | | **Patients Are Getting Better on Antibiotics:** Participants describe how providers don't want to stop antibiotics because the patients are getting better |
|  | **Virtual Examinations:** Participants describe how ID was choosing to do remote exams | |
| **Task Challenges** | | |
|  | **Limited Prospective Interventions:** Participants talk about how there aren't a lot of interventions aimed at stopping the first does of antibiotics, most are focused on de-escalating or stopping once started and how that can be a limitation of AMS | |
|  | **Manual Tasks:** Participants describe how a lot of the AMS work is manual so is impacted by being sick etc. | |
|  | **Rapid Decision Making:** Participants talk about how the need for rapid decision making can get in the way of AMS | |
|  | **Therapeutic Momentum:** Participants describe how it can be hard to stop antibiotics once they are started, or how physicians want to finish a course of antibiotics because there are just a few days left (often discussed in relation to cultures coming back) | |
|  | | **General De-escalation:** Participants describe challenges with de-escalation in general without giving an explicit reason why. |
|  | **Workload:** Description of how workload impacts ability to do AMS activities. Includes comments like, I only have a few minutes to see a patient. Or the recommendation gets lost in the 100s of orders | |
|  | | **Competing Priorities:** Participants talk about how AMS isn't the only priority |
| **Tools and Technology Challenges** | | |
|  | **Challenges with Cultures:** Participants describe issues with contaminated cultures and that impacting AMS. Would include difficulties obtaining cultures due to intubation or being on BiPap | |
|  | **Challenges with Procalcitonin:** Participants describe how providers weren't using procal correctly, overutilization or underutilization or they couldn't use it because they didn't have the testing infrastructure | |
|  | **Changes in Guidelines or Knowledge about a Disease State:** Changes in guidelines that impact AMS | |
|  | **No Specific Guidance for Antibiotics:** Participants talk about how the guidance, mainly COVID-19 guidance, was not specifically focused on antibiotics or how there was an absence of recommendations for antibiotics in COVID-19 guidance. This would include antibiotics being part of the recommended treatment | |
|  | **Supply/Drug Shortages:** Participants describe not having adequate supplies, could be PPE or even drug shortages | |
|  | **Testing Delays:** Participants describe delays with testing or shortages of COVID-19 tests. Would include other challenges with COVID-19 testing such as extremely rigid criteria for sending tests | |
| **Strategies that Support AMS** | | |
| **External Factors that Promote AMS** | | |
|  | **External Literature or Studies:** Participants describe how external literature, guidelines or studies impact AMS. Would include making decisions based on existing literature | |
|  | **Look Outside Your Institution:** Participants describe modeling what you do based on other institutions | |
|  | **National Guidance:** Participants describe the value of national guidance either from the CDC or NIH | |
|  | **Outside Consulting:** Participants describe using outside consulting services to promote AMS | |
|  | **Statewide Collaborative:** Participants describe statewide collaboratives or other external groups that support/facilitate AMS; would also include community initiatives at a smaller level. Key for this code is it needs to be collaborations across healthcare systems not within systems | |
| **Internal Environment Strategies** | | |
|  | **Accessible:** Participants describe accessibility as a feature of AMS, co-location or open door policy or whatever. Includes comments about being visible | |
|  | **Location of Pharmacist of AMS Team:** Participants describe how location of the team is a strategy for AMS | |
| **Organization: AMS Program Structure** | | |
|  | **AMS Resources:** Participants describe how the resources devoted to AMS impact what they can or cannot do for AMS | |
|  | | **Staffing:** Participants describe how increases/decreases in staffing impacted AMS |
|  | **Decentralized AMS:** Participants describe how AMS is the responsibility of every provider or every pharmacist, and isn't centralized in one person | |
|  | **Division of Responsibilities:** Strategic division of responsibilities to promote AMS | |
|  | | **Best People Managed COVID-19:** Participants describe how the ‘best’ people (providers, pharmacists, nurses, etc.) managed COVID-19 either by request or because they volunteered |
|  | | **Trainee Responsibilities:** Participants talk about the role trainees had in promoting AMS |
|  | **Focus of AMS Program or Change in Focus:** Participants describe having to change their focus of the AMS program; would also include comments of COVID-19 is just a new reality for us. COVID-19 is here to stay. Also includes how they decide what to focus on for AMS | |
|  | **System-wide AMS:** Participants describe protocols across multiple sites or coordination of AMS activities across multiple sites | |
| **Organization: Hospital Level Factors that Impact AMS** | | |
|  | **Culture of AMS:** Participants describe the culture of AMS at their hospital or in their system; would also include participants describing history of AMS or a good track record of AMS at their institution. Includes when they talk about acceptance rate of interventions. Would include mentions of strong AMS leadership | |
|  | **Employment Model:** Providers describe how having multiple provider groups employed by different methods impacts effectiveness of AMS | |
|  | **Flexibility:** Participants describe how hospital flexibility or agility or ability to make decisions quickly impacts AMS | |
|  | | **Changes in Hospital Procedures or Patient Population:** Participants describe how hospital procedures changed due to COVID-19 or how patient population changed due to COVID-19 |
|  | | **Constant Adaptation:** Participants describe how they constantly have to adapt to promote AMS |
|  | | **New Spaces:** Participants describe opening new spaces or even a new hospital to help manage during COVID-19 or having COVID-19 only wards |
|  | **Hospital-wide/System Leadership:** Participants describe how hospital level or system leadership impacts AMS or how hospital wide mandates impact AMS | |
|  | **Hospital Culture:** Description of how the hospital culture (not the AMS culture) promotes AMS. We are a center of excellence in all we do etc. | |
|  | **Hospital Size:** Participants describe how hospital size impacts AMS. Mostly how being small helps AMS | |
|  | **Staff Appreciation and Support:** Participants describe how staff appreciation or support promotes AMS; could be thank days or praising staff for the work they do | |
|  | **Standardization:** Participants describe ways in which they try to standardize antibiotic use, and how this promotes AMS | |
|  | **Trust in Decision-Making of Providers:** Participants talk about how there must be trust in the decision making of providers | |
| **Organization: Promoted Collaboration** | | |
|  | **Communication with Providers:** Participants describe how they communicate with physicians or strategies they use to communicate to promote AMS; would also include descriptions of where providers can access guidelines, such as an intranet. Also includes when they talk about the utility of peer to peer conversations | |
|  | **Educating Providers:** Description of how education of providers promotes AMS | |
|  | **Frequent Collaboration:** Participants describe how meeting frequently or frequent contact facilitates AMS | |
|  | **In-person Stewardship Activities:** Participants describe how face-to-'face stewardship promotes AMS. Includes face to face stewardship conversations. Could be handshake stewardship. Includes when the participants talk about a desire to have been more present during COVID-19 or rounding with ID team; this would be co-coded with accessible | |
|  | **Multidisciplinary Team:** Participants describe the multidisciplinary nature of their AMS group. Could include what they do to promote buy in with their guidelines. Includes making someone a physician champion to promote buy in | |
|  | **Prior AMS Initiatives:** Participants describe how prior work in an area helped acceptance of AMS recommendations, e.g., viral pneumonia, helped with stopping antibiotics for COVID-19 | |
|  | **Relationship with Providers:** Participants talk specifically about what they do to enhance their one on one relationships with providers. Most often, this is about acceptance or recommendations but not exclusively. It includes comments about having rapport or building rapport with physicians | |
|  | **Teamwork Within Your Team:** Participants describe how working together promotes AMS. This could be within their clinical team or across teams. This includes comments about sharing the load. This differs from relationships with other providers because this is more of a team based code. Relationship with providers is about how individual relationships impact AMS | |
|  | **Workgroup:** Participants describe workgroups they have to facilitate AMS, whether that was for COVID-19 or for AMS broadly, both are okay | |
| **Organization/Person: ID Leadership or Engagement** | | |
|  | **ID Consults to Promote AMS:** Participants describe how ID consults are utilized to promote AMS. Could be Pharmacy or Physician consults | |
|  | **ID Expertise to Promote AMS:** Participants describe how ID expertise impacts AMS. Would include participating in rounds | |
|  | | **In Person Examination:** Participants talk about the value of in person examinations in the diagnostic/treatment process |
|  | **ID Responsibility for Managing Infectious Diseases:** Participants describe ID responsibilities managing infectious disease. A lot of times this was specific to COVID-19 patients. At some sites ID saw all COVID-19 patients. At some sites there were other strategies for management. | |
| **Person (Healthcare Staff) Characteristics that Impact AMS** | | |
|  | **AMS Emphasized More in Training:** Participants describe how AMS being emphasized more in training promotes better AMS | |
|  | **Did the Best We Can:** Participants describe doing the best they can with what resources they had or the personnel they had, etc. | |
|  | **Just in Case Antibiotics:** Participants describe giving antibiotics to cover their bases. Would include treating empirically once a COVID-19 test came back negative | |
|  | **Learning from Experience:** Participants describe how learning from experience, particularly related to COVID-19 but also with other viral diseases impacted AMS | |
|  | **Length of Time at a Site or System:** Participants describe how length of time at a site impacts AMS. Would also include comments about the importance of retaining staff | |
|  | **Personal Commitment of the Staff**: Participants describe how personal characteristics, dedication etc. impact AMS. Also includes having excellent people in the right places | |
|  | **Provider Beliefs or Preferences:** Participants describe how provider beliefs get in the way of or help AMS. Includes comments about willingness to accept recommendation | |
|  | **Trying to Keep People Alive:** Participants say things like we were just trying to keep people alive, we threw the kitchen sink at them, etc. | |
| **Person (Patient) Characteristics that Impact AMS** | | |
|  | **Educating Patients:** Participants talk about needing to educate patients to promote AMS | |
| **Task Strategies that Impact AMS** | | |
|  | **Consistency with AMS Work**: Recognition that it is about consistency and persistence and if we stay focused and consistent then AMS goals will improve | |
|  | **Increase in Hours:** Increased hours of staff to promote AMS. Could also include being on call in a way that was different during COVID-19 | |
|  | **Preparedness:** Whether this is being prepared for meetings or in other ways. Includes observing experiences of others and preparing based on their experiences if the pandemic came later to their site | |
|  | **Prioritization:** Participants describe how they prioritize AMS activities; could also be prioritizing which patients got treatments etc. | |
|  | **Proactive in Responding/Monitoring:** Participants describe how if they are on their game, or on top of it, they can stop antibiotics | |
|  | **Small Victories/Incremental Change:** Participants describe negotiating with the guidelines or accepting small victories regarding AMS. They wouldn't go along with stopping antibiotics for all patients, but they would stop for mild cases | |
| **Tools and Technology: AMS Tests** | | |
|  | **Chest Imaging:** Participants describe how chest imaging can help promote AMS most often how it can or cannot help with distinguishing bacterial and viral infections | |
|  | **COVID-19 Test:** Participants describe how the COVID-19 Test helped with AMS, most often as it relates to diagnostic uncertainty | |
|  | **Procalcitonin:** Participants describe how procalcitonin facilitated AMS | |
|  | **Viral Panel:** Participants described how a viral panel was useful in promoting AMS | |
| **Tools and Technology: AMS Tools** | | |
|  | **Antibiogram:** Participants describe how antibiograms or susceptibility reports guide AMS | |
|  | **Antibiotic Timeouts:** Antibiotic time outs, or when they talk about evaluating appropriateness after a set time frame, 24h or 48h usually | |
|  | **Automated Flags:** Participants talk about how the automated flags can help with prioritization or other aspects of AMS | |
|  | **Automatic Involvement:** Participants describe how the AMS or ID teams can automatically involve themselves in specific cases | |
|  | **Benchmarks:** Participants describe how they use benchmarking, comparing within their organization or outside, to promote AMS | |
|  | **Clinical Culture Review:** Participants describe how they review culture results to promote AMS. Includes utilization of cultures to narrow spectrum | |
|  | **COVID-19 Therapeutics:** Description of how having COVID-19 Therapeutics available promoted AMS | |
|  | **Delegation Protocols:** Participants describe how delegation protocols are used in AMS; this would include pharmacy driven interventions to reduce antibiotics | |
|  | **EHR Features:** Participants describe using dot phrases, clinical decision support or other EHR features to promote AMS | |
|  | | **EHR Reports:** Participants describe how EHR reports can be helpful with AMS |
|  | | **Indication Requirements:** Participants describe how requiring providers to indicate the disease they are treating when selecting antibiotics is helpful in AMS |
|  | | **Order Sets:** Participants describe how order sets are used to promote AMS |
|  | **Guidelines or Protocols:** Participants describe developing guidelines or protocols; includes formal or informal guidelines or guidance to promote AMS; includes bundles | |
|  | **Pre-Approval of Antibiotics:** Participants describe requiring pre-approval of antibiotics; could also be unofficial consultation before first dose; or discussion of the idea of stopping antibiotics before they are started (momentum) | |
|  | | **Restricted Formulary:** Participants describe having a restricted formulary, only certain people can order certain drugs, or you are required to have an ID consult. |
|  | **Telehealth:** Participants were able to see patients remotely | |
| **Tools and Technology: AMS Tracking** | | |
|  | **Antibiotic Prescribing Reports:** Could be when they manually run reports to get lists of patients on antibiotics or physician report cards or lists of top prescribers, things like that | |
|  | **Pay for Performance:** Participants describing basing compensation off AMS metrics; would include penalties at the hospital level | |
|  | **Prospective Audit and Feedback:** Participants describe using prospective audit and feedback to promote AMS | |
| **Miscellaneous** | | |
| **Interesting Quotes:** Quotes made by the participants that are descriptive or worth noting that may potentially be used to convey important aspects of the diagnosis and treatment of infection process, potentially in a publication | | |
| **Talking about Experiences of Others:** Use this tag if you feel like a participant is really extrapolating a little too far; speaking beyond their own experiences. | | |
| **Abbreviations:** AMS: Antimicrobial Stewardship; EHR: Electronic Health Record; ID: Infectious Diseases | | |
