# Supplementary material for: Optimizing antimicrobial stewardship during operational upheaval: lessons in resiliency from the COVID-19 pandemic
Source: Infect Control Hosp Epidemiol. 2026 Mar 26;47(5):499–508. doi: 10.1017/ice.2026.10415 (PMC13040247; doi:10.1017/ice.2026.10415)
Supplement: Schwei et al. supplementary material 2 — Schwei et al. supplementary material [file S0899823X26104152sup002.docx]

**CARB R01 Semi-structured Interview Guide for Pharmacists**

Thank you for taking the time to speak with us today. I am part of a team of researchers from University of Wisconsin trying to understand the impact of COVID-19 on antibiotic prescribing in acute care settings.

I am looking forward to having an open, honest and interactive discussion. We appreciate any feedback you have, as it is essential to helping us understand how your hospital was impacted by the COVID-19 pandemic. Please formulate your responses based on your experiences at your primary hospital if you work at more than one. If at any point you are unclear what we are asking about please ask us.

I will be recording our discussion so that we are able to review it for subsequent analysis. No one outside of the research team will listen to these recordings. Your comments will be anonymous and we will never use your name or other information that would make it possible to identify you outside of this conversation. Do you have any questions or any concerns about recording this discussion?

To start, I have a few demographic questions:

1. What is your current position including any titles?
2. What licenses or certifications do you have currently?
3. How many years of post-training experience do you have (include residency + fellowship as training years):
4. How many years have you been at your current hospital:
5. What is your biological sex?

Shall we begin?

1. Can you describe your hospital’s antibiotic stewardship program before COVID? Team members and structure? Roles? Primary activities?
   1. [How much of your antimicrobial stewardship work is focused on the ICU vs. inpatient wards vs. ED vs. ambulatory]
      1. What initiatives do you have in the ED
   2. Please describe core components of your stewardship program?
   3. Do you do any handshake stewardship? If so, please describe
   4. We’re very interested in respiratory conditions in general, both COVID and pneumonia. Can you tell us a little bit about any antibiotic stewardship interventions you have specifically for pneumonia or other respiratory conditions? [diagnostic stewardship, tailoring therapy, de-escalation]
      1. Did you focus at all on distinguishing viral and bacterial respiratory diseases pre-pandemic
2. Can you walk us through the various phases of COVID and how it impacted the roles of infectious disease pharmacists and antibiotic stewardship programs at your hospital?
   1. Based on response try to probe on any personal, environmental, organizational, technological changes that were happening
3. As a hospital, how did antibiotic prescribing for patients with suspected or confirmed COVID evolve from the beginning of COVID to now?
   1. What do you think were the things driving antibiotic prescribing in the early phase? [severity of disease, diagnostic uncertainty, availability of treatment options, availability of testing]
   2. How did COVID diagnostics, including their availability and turn around time evolve throughout the pandemic? [who was running test, county vs. in-house]
   3. Did surges throughout the pandemic influence antibiotic stewardship activities?
4. Were there any tools available at your hospital to help guide providers on when to prescribe antibiotics for patients being evaluated for potential COVID? [institutional guidance] When was the first guidance released? How has it evolved throughout the pandemic? Who issues the guidance? What specific diagnostic tests were available [availability/speed of testing], [use of procalcitonin], [tools and technology and organization] ? How has this evolved throughout the pandemic? Has the availability of the tests impacted antibiotic prescribing behavior or guidance?
   1. Were there any ED specific interventions
5. How did your hospital’s antibiotic stewardship program evolve during COVID? Change in approaches? Prioritization of activities? Challenges? Innovations?
   1. If did not evolve, ask how they were able to maintain programming given the added responsibilities of covid.
   2. When times got tough, what were the antibiotic stewardship practices that fell by the wayside? What types of activities were pushed to the side?
6. Recent data suggests a majority of inpatients with COVID still receive antibiotics, do you still observe this? If so, what do you think is driving this? [diagnostic uncertainty] [coinfection], [person]
7. What makes an antibiotic stewardship program resilient in the face of operational stress- stress from a new disease that we don’t understand, stress from high volumes of patients etc. what do you think your program did particularly well to stay resilient in your stewardship goals as a program?’
8. Looking back, are there things that your hospital could have done better if there had been no resource limitation, etc.?
9. Is there anything else you think we should know about antibiotic prescribing at your institution related to the COVID-19 pandemic?

**CARB R01 Semi-structured interview guide for Physician Interviews**

Thank you for taking the time to speak with us today. I am part of a team of researchers from University of Wisconsin trying to understand the impact of COVID-19 on infection control and antibiotic prescribing in acute care settings.

I am looking forward to having an open, honest and interactive discussion. We appreciate any feedback you have, as it is essential to helping us understand how your hospital was impacted by the COVID-19 pandemic. Please formulate your responses based on your experiences at your primary hospital if you work at more than one. If at any point you are unclear what we are asking about please ask us.

I will be recording our discussion so that we are able to review it for subsequent analysis. No one outside of the research team will listen to these recordings. Your comments will be anonymous and we will never use your name or other information that would make it possible to identify you outside of this conversation. Do you have any questions or any concerns about recording this discussion?

To start, I have a few demographic questions:

1. What is your current position including any titles?
2. What board certifications do you have currently?
3. How many years of post-training experience do you have(include residency + fellowship as training years):
4. How many years have you been at your current hospital:
5. Has your institution or position changed since 2019, if so what was your prior position and timeframe?
6. What is your biological sex?

Shall we begin?

1. Can you describe your hospital’s antibiotic stewardship program before COVID? Team members and structure? Roles? Primary activities?
   1. [How much of your antimicrobial stewardship work is focused on the ICU vs. inpatient wards vs. ED vs. ambulatory]
      1. What initiatives do you have in the ED
   2. Please describe core components of your stewardship program?
      1. Any initiatives for respiratory infections? Viral vs. Bacterial disease?
   3. As an ID physician, what does your day-to-day work look like for AMS?
      1. Are ID consults well utilized/under utilized? Helpful for AMS
2. Can you walk us through the various phases of COVID and how it impacted clinical operations in your emergency department and hospital?
   1. How frequently were ID consults used at the beginning of the pandemic
3. Walk me through your personal approach to antibiotic prescribing decisions for patients with suspected COVID (symptoms but no confirmatory test) during this time period when rapid testing was unavailable? How, if at all, has this evolved from early in the pandemic?
   1. Were there any tools that helped you decide when to prescribe antibiotics in this population during the early months of the pandemic? Did you hospital have any guidelines or care pathways for this population? If so, did they include antibiotics? [availability/speed of testing], [use of procalcitonin], [tools and technology and organization]
   2. What do you think were the things driving antibiotic prescribing in the early phase? [severity of disease, diagnostic uncertainty, availability of treatment options, availability of testing]
4. As a hospital, how did antibiotic prescribing for patients with suspected or confirmed COVID evolve from the beginning of COVID to now?
   1. How did COVID diagnostics, including their availability and turn around time evolve throughout the pandemic? [who was running test, county vs. in-house]
5. Please describe how the pandemic influenced antibiotic stewardship programs and antibiotic prescribing overall (beyond just for patients with suspected or confirmed COVID)? Did this change based on number of cases or surges?
   1. Were you able to maintain routine AMS activites
      1. If yes, how were you able to maintain everything given the new responsibilities
   2. What fell by the wayside when things got tough?
6. Recent data suggests a majority of inpatients with COVID still receive antibiotics, do you ever prescribe antibiotics to patients with confirmed COVID? If so, how do you make that decision? [diagnostic uncertainty], [person]
7. What makes an antibiotic stewardship program resilient in the face of operational stress- stress from a new disease that we don’t understand, stress from high volumes of patients etc. what do you think your program did particularly well to stay resilient in your stewardship goals as a program?’
8. Looking back, are there things that your hospital could have done better if there had been no resource limitation, etc.?
9. Is there anything else you think we should know about infection control/prevention and antibiotic prescribing at your institution related to the COVID-19 pandemic?
